# Leukotriene B_4_ receptor type 2 protects against pneumolysin-dependent acute lung injury

**DOI:** 10.1038/srep34560

**Published:** 2016-10-05

**Authors:** Misako Shigematsu, Tomoaki Koga, Ayako Ishimori, Kazuko Saeki, Yumiko Ishii, Yoshitaka Taketomi, Mai Ohba, Airi Jo-Watanabe, Toshiaki Okuno, Norihiro Harada, Takeshi Harayama, Hideo Shindou, Jian-Dong Li, Makoto Murakami, Sumio Hoka, Takehiko Yokomizo

**Affiliations:** 1 Department of Biochemistry, Juntendo University School of Medicine, Tokyo, Japan; 2 Department of Anesthesiology and Critical Care Medicine, Graduate School of Medical Sciences, Kyushu University, Fukuoka, Japan; 3 Department of Respiratory Medicine, Juntendo University School of Medicine, Tokyo, Japan; 4 Research Institute for Diseases of the Chest, Graduate School of Medical Sciences, Kyushu University, Fukuoka, Japan; 5 Lipid Metabolism Project, Tokyo Metropolitan Institute of Medical Science, Tokyo, Japan; 6 Lipid Signaling Project, National Center for Global Health and Medicine, Tokyo, Japan; 7 Department of Biochemistry, University of Geneva, Geneva, Switzerland; 8 Center for Inflammation, Immunity and Infection, Institute for Biomedical Sciences, Georgia State University, Atlanta, GA, USA.

## Abstract

Although pneumococcal infection is a serious problem worldwide and has a high mortality rate, the molecular mechanisms underlying the lethality caused by pneumococcus remain elusive. Here, we show that BLT2, a G protein-coupled receptor for leukotriene B_4_ and 12(S)-hydroxyheptadecatrienoic acid (12-HHT), protects mice from lung injury caused by a pneumococcal toxin, pneumolysin (PLY). Intratracheal injection of PLY caused lethal acute lung injury (ALI) in BLT2-deficient mice, with evident vascular leakage and bronchoconstriction. Large amounts of cysteinyl leukotrienes (cysLTs), classically known as a slow reactive substance of anaphylaxis, were detected in PLY-treated lungs. PLY-dependent vascular leakage, bronchoconstriction, and death were markedly ameliorated by treatment with a CysLT1 receptor antagonist. Upon stimulation by PLY, mast cells produced cysLTs that activated CysLT1 expressed in vascular endothelial cells and bronchial smooth muscle cells, leading to lethal vascular leakage and bronchoconstriction. Treatment of mice with aspirin or loxoprofen inhibited the production of 12-HHT and increased the sensitivity toward PLY, which was also ameliorated by the CysLT1 antagonist. Thus, the present study identifies the molecular mechanism underlying PLY-dependent ALI and suggests the possible use of CysLT1 antagonists as a therapeutic tool to protect against ALI caused by pneumococcal infection.

Pneumonia is a major cause of morbidity and mortality worldwide^1^. *Streptococcus pneumoniae (i.e.*, pneumococcus) is the capital pathogen responsible for community-acquired pneumonia^2^. Although various antibiotics have been developed and used to treat patients, the mortality of patients with severe pneumococcal infections remains unacceptably high, particularly during the early phase^3^,^4^,^5^. Thus, novel therapeutic strategies should be developed based on a full understanding of molecular pathogenesis of lethal pneumococcal infections. The pneumococcal toxin pneumolysin (PLY), a 53 kDa cytoplasmic protein, is a key virulence factor with pore-forming activity against target membranes^6^. PLY released by pneumococcus causes direct injury in airway epithelial cells and pulmonary vascular cells, leading to acute lung injury (ALI) during the initial phase^7^. PLY-dependent pneumococcal invasion into the bloodstream results in pneumococcal bacteremia and sepsis during the later phase^8^. PLY is produced by all clinical isolates of pneumococcus, and PLY-deficient pneumococcal strains show reduced virulence in animal models^6^. Therefore, controlling PLY-induced ALI is a novel therapeutic strategy against severe pneumococcal infection, and can be used as an adjunct to existing therapies such as antibiotics and mechanical respiratory support^9^,^10^, particularly during the acute phase of infection.

Leukotriene B_4_ (LTB_4_) receptor type 2 (BLT2) is a class A G protein-coupled receptor (GPCR). It was originally identified in our laboratory as a low-affinity receptor for LTB_4_, which is produced from arachidonic acid (AA) by 5-lipoxygenase (5-LO) and LTA_4_ hydrolase^11^; however, we later found that another AA metabolite 12(*S*)-hydroxyheptadeca-5*Z*,8*E*,10*E*-trienoic acid (12-HHT), which is produced downstream of cyclooxygenases (COXs), is the high-affinity endogenous ligand of BLT2^12^. We previously showed that BLT2 is expressed in intestinal epithelial cells and skin keratinocytes, and that 12-HHT/BLT2 signaling maintains intestinal barrier function and accelerates skin wound healing^13^,^14^. Recently, we found that BLT2 expressed in keratinocytes enhances the expression of several tight junctional proteins, including claudin 4, and protects against invasion by foreign antigens^15^. Taken together, these data suggest a crucial role for BLT2 in epithelial barrier homeostasis.

Based on the finding that BLT2 is expressed in pulmonary epithelial cells and vascular endothelial cells in the mouse lung, we hypothesized that BLT2 may play a protective role in lung epithelia. Thus, we treated mice with PLY to induce ALI and explored the physiological role of BLT2 in the lung.

## Results

### BLT2-knockout mice are highly susceptible to acute lung damage upon intratracheal administration of PLY

**Figure 1:**
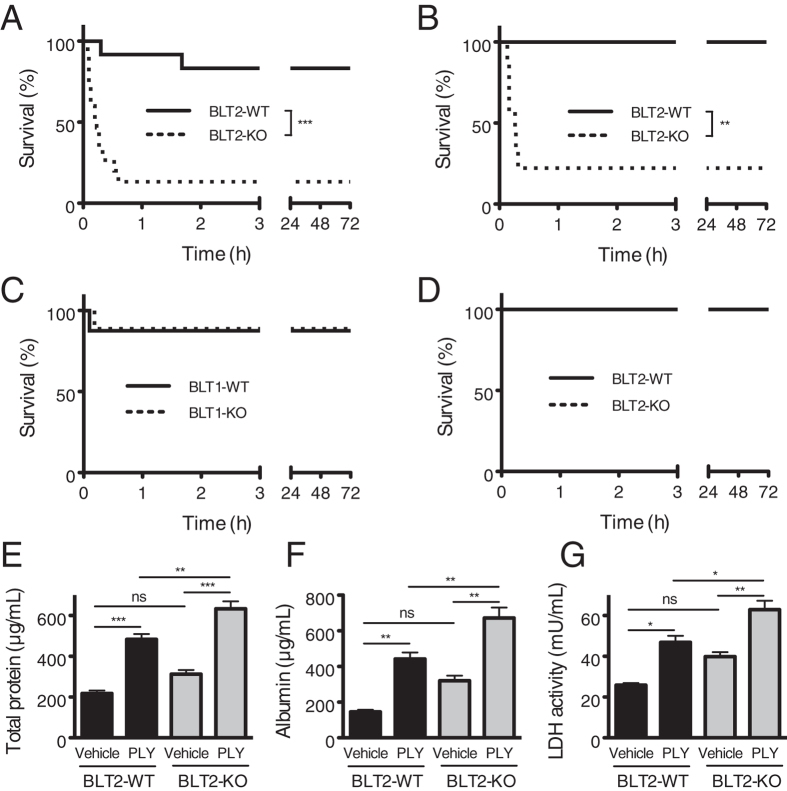
BLT2-deficient mice are more susceptible to lung damage induced by intratracheal administration of PLY. (**A**–**D)** Mice received 50 ng of PLY and survival was monitored for 72 h. BLT2-WT and BLT2-KO mice on a BALB/c background (**A,D**) or on a C57BL/6 background (**B**), and BLT1-WT and BLT1-KO mice on a BALB/c background (**C**) were subjected to PLY administration intratracheally (**A–C**) or intravenously (**D**). Kaplan-Meier curves are shown for each experimental condition. Data from multiple experiments were pooled (n = 8–15). Data were analyzed using the log-rank test: ***p < 0.0001; **p = 0.0014. (**E–G**) BLT2-WT and BLT2-KO mice on a BALB/c background were intratracheally administered 50 ng of PLY or saline (vehicle control). BAL was performed 10 min later. The total protein concentration (**E**), the albumin concentration (**F**), and LDH activity (**G**) were measured in BAL fluid (n = 4 for vehicle control; n = 8–10 for PLY treatment). Data were analyzed by one-way ANOVA, followed by the Tukey’s *post-hoc* test: ns, not significant; *p < 0.05; **p < 0.01; ***p < 0.001.

To investigate the physiological function of BLT2 in the lung, BLT2-knockout (BLT2-KO) and the littermate control wild-type mice (BLT2-WT) on BALB/c (Fig. 1A) and C57BL/6 (Fig. 1B) backgrounds were intratracheally injected with 50 ng of recombinant PLY. Surprisingly, about 80% of BLT2-KO mice on both backgrounds died within 10 to 30 min after PLY administration, in clear contrast to BLT2-WT mice (Fig. 1A,B). As BLT1^16^ and BLT2 are highly homologous, we repeated the experiment using BLT1-knockout (BLT1-KO) and the littermate control wild-type (BLT1-WT) mice and found that BLT1-KO mice were not susceptible to the effects of intratracheal PLY administration (Fig. 1C). Furthermore, intravenous injection of PLY did not cause death at all (Fig. 1D), showing that systemically administrated PLY is not lethal by itself; rather, it causes topical injury and inflammation upon local administration in the lungs.

Thus, we next examined PLY-induced acute lung damage by measuring the concentrations of total protein and albumin, along with LDH activity, in bronchoalveolar lavage (BAL) fluid. All of these parameters were increased after PLY administration, even in BLT2-WT mice; however, values were significantly higher in BLT2-KO mice, which showed more severe lung damage (Fig. 1E–G). Taken together, these results suggest that BLT2-KO mice are more susceptible to lung damage caused by intratracheal PLY administration, regardless of their genetic background.

### BLT2 is expressed in alveolar epithelial type II and vascular endothelial cells in the mouse lung

**Figure 2:**
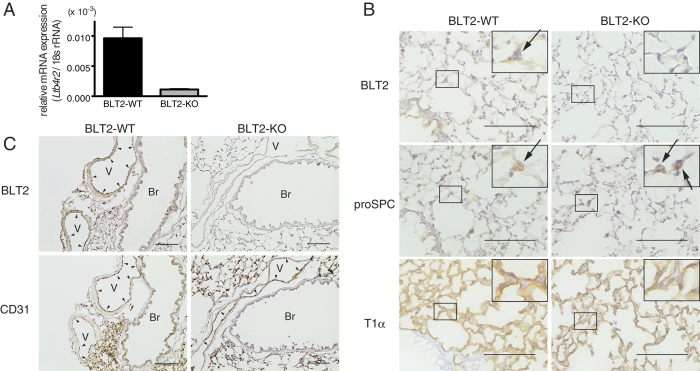
BLT2 is expressed in type II alveolar epithelial cells and vascular endothelial cells in mouse lung. (**A**) Expression of BLT2 mRNA in BLT2-WT and BLT2-KO mouse lungs as measured by q-PCR (n = 8). (**B**) BLT2 expression was compared with that of proSP-C and T1α. The upper right insets in each panel show higher magnifications of the boxed areas. Arrows indicate AT II cells. (**C**) BLT2 expression was compared with that of CD31. Arrowheads indicate vascular endothelial cells. V: vessel, Br: bronchus. Scale bars: 100 μm.

BLT2 is mainly expressed in tissues exposed to the external environment, such as skin and intestine^17^. We previously reported that BLT2 protects mouse intestinal epithelial cells against colitis^14^, and that BLT2 expression in keratinocytes is important for accelerating skin wound healing^13^. Because no study has examined expression of BLT2 in the lung, we next investigated whether BLT2 is expressed in mouse lung by detecting BLT2 mRNA by quantitative PCR (Fig. 2A). To identify BLT2-expressing cells in mouse lung, we performed immunohistochemical analysis of serially sectioned lung tissues obtained from BLT2-WT and BLT2-KO mice. BLT2 signals were detected in proSP-C-positive alveolar epithelial type II (AT II) cells, but not in T1α-positive alveolar epithelial type I (AT I) cells (Fig. 2B). BLT2 was also expressed in CD31-positive lung vascular endothelial cells (Fig. 2C). No BLT2 signals were detected in AT II cells and vascular endothelial cells in BLT2-KO mice, confirming the specificity of the anti-mouse BLT2 antibody.

### BLT2 deficiency augments PLY-induced vascular hyperpermeability and bronchoconstriction

**Figure 3:**
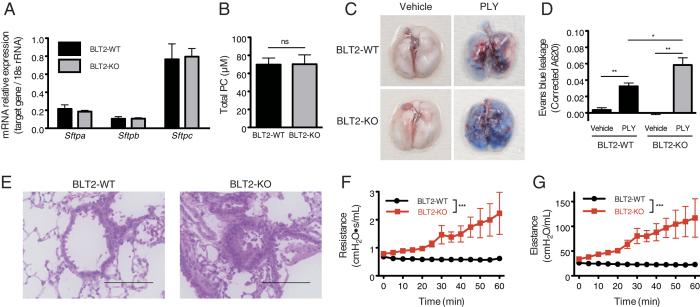
BLT2 deficiency exacerbates vascular permeability and bronchoconstriction after PLY treatment. (**A**) Alveolar type II cells were isolated from BLT2-WT and BLT2-KO mouse lungs, and mRNA for surfactant protein (**A–C**) was assessed by quantitative real time-PCR (n = 8). (**B**) The concentrations of phosphatidylcholine in the BAL fluid from BLT2-WT and BLT2-KO mouse were measured in an enzyme-based fluorescent assay (n = 4–5). (**C**,**D**) Mice were intravenously injected with 0.5% Evans blue 30 min prior to intratracheal administration of PLY. Accumulation of Evans blue in tissues was calculated using the following formula: Corrected A_620_ = A_620_ − (1.426 × A_740_ + 0.03). Representative images (**C**) and quantification (**D**) are shown (n = 3 for vehicle control; n = 13–16 for PLY treatment). Data were analyzed by one-way ANOVA, followed by the Tukey’s *post-hoc* test: *p < 0.05. (**E**) Representative images of H&E-stained lung sections from PLY-treated BLT2-WT and BLT2-KO mice. Scale bars: 100 μm. (**F**,**G**) Mice were treated with PLY and mechanically ventilated. The airway resistance (**F**) and the elastance (**G**) were measured every 5 min for 60 min (n = 6). Data were analyzed by two-way ANOVA: ***p < 0.0001.

We next investigated the mechanisms underlying the PLY-induced death of BLT2-KO mice with respect to the function of AT II cells and vascular endothelial cells. One of the important functions of AT II cells is to produce and secrete pulmonary surfactant; therefore, we examined whether BLT2 deficiency affects surfactant production. To do this, we measured the expression of mRNAs for surfactant proteins in isolated AT II cells, and the concentration of phosphatidylcholine (PC), the major surfactant lipid, in BAL fluid from BLT2-WT and BLT2-KO mice^18^. The levels of surfactant protein A, B and C mRNA (*Sftpa*, *Sftpb*, and *Sftpc*, respectively) in isolated AT II cells were not affected by BLT2 deficiency (Fig. 3A). The number of AT II cells was also similar in BLT2-WT and BLT2-KO mice (data not shown). The concentration of PC in BAL fluid from BLT2-WT and BLT2-KO mice was also similar (Fig. 3B). These results indicate that BLT2 deficiency has no effect on AT II cell function, at least in terms of surfactant production.

Because a previous study reported PLY-induced lung vascular leakage during the early phase of infection^19^, we next examined endothelial function in BLT2-WT and BLT2-KO mice. Intratracheal administration of PLY caused leakage of Evans blue from vasculature in both BLT2-WT and BLT2-KO mice, but it was more obvious in BLT2-KO mice (Fig. 3C). Quantification of the leaked dye clearly showed that PLY-dependent vascular leakage was significantly higher in BLT2-KO mice than in BLT2-WT mice (Fig. 3D). Examination of hematoxylin and eosin (H&E)-stained lung sections showed that, in addition to vascular leakage, BLT2-KO mice developed severe bronchoconstriction upon PLY administration (Fig. 3E). However, PLY did not induce bronchoconstriction in BLT2-WT mice (Fig. 3E). Thus, we next assessed pulmonary function using the flexiVent. We found that both airway resistance and elastance in BLT2-KO mice were higher after PLY administration; these effects were time-dependent and were not observed in BLT2-WT mice (Fig. 3F,G). Airway resistance is a quantitative measure of airflow limitation, and elastance reflects the rigidity of the lung tissue. The observed increase in both parameters in BLT2-KO mice might account for the bronchoconstriction and the vascular hyperpermeability induced by PLY. Taken together, these data suggest that BLT2 deficiency causes severe vascular leakage and bronchoconstriction, but has no effect on the production of pulmonary surfactant.

### PLY triggers the production of cysteinyl leukotrienes

**Figure 4:**
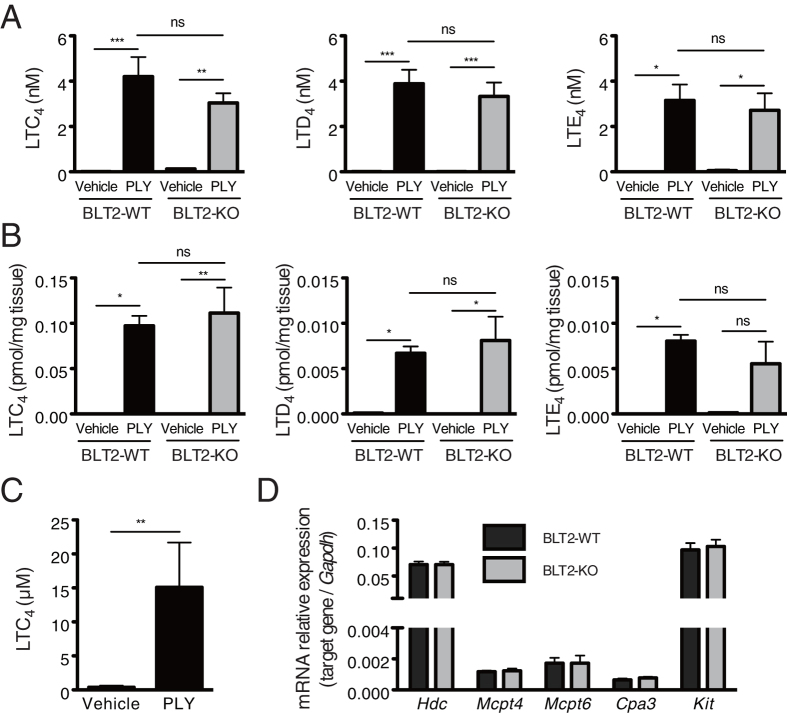
PLY treatment induces production of cysteinyl leukotrienes in both BLT2-WT and BLT2-KO mouse lungs. (**A**,**B**) Mice received PLY or vehicle intratracheally, and BAL fluid (**A**) and lung tissue (**B**) were harvested 10 min later. The concentrations of LTC_4_, LTD_4_, and LTE_4_ were measured by LC-MS/MS. (**A**) expressed as nM in BAL fluid (n = 6–11); (**B**) expressed as pmol per mg tissue (n = 4). Data were analyzed by one-way ANOVA, followed by the Tukey’s *post-hoc* test: ns, not significant; *p < 0.05; **p < 0.01; ***p < 0.001. (**C**) BMMCs were stimulated with 50 ng/mL PLY for 5 min. The concentration of LTC_4_ in the supernatant was measured by LC-MS/MS (n = 6). Data were analyzed using Student’s *t* test: **p < 0.01. (**D**) Expression of mRNA for mast cell markers in BLT2-WT and BLT2-KO mouse lung, as measured by quantitative real time-PCR (n = 4).

The vascular hyperpermeability and bronchoconstriction observed in BLT2-KO mice after PLY administration resemble a lethal asthma attack in a human patient; thus, we hypothesized that these characteristics may be linked to bronchoconstrictors such as cysteinyl leukotrienes (cysLTs; leukotriene C_4_ (LTC_4_), leukotriene D_4_ (LTD_4_) and leukotriene E_4_ (LTE_4_)), collectively known as slow reactive substance of anaphylaxis (SRS-A)^20^. Thus, we next measured the eicosanoid content of the BAL fluid and lung tissue from PLY-treated BLT2-WT and BLT2-KO mice using LC-MS/MS (Fig. 4A,B and Table 1)^21^. Surprisingly, PLY administration led to the production of large amounts of cysLTs in the BAL fluid and lung tissue, which were almost undetectable in vehicle-treated mice (Fig. 4A,B). PLY also increased the levels of other eicosanoids (LTB_4_, 12-HETE) in BAL fluid and lung tissue (Table 1), although there were present at much lower levels than cysLTs. Basal levels of prostaglandin (PG) and 12-HHT were detectable, but were not affected by PLY (Table 1). PLY treatment increased the concentration of cysLTs in diluted BAL fluid to about 4 nM, suggesting that high concentrations of cysLTs are produced in the lung after PLY administration, followed by subsequent activation of three receptors for cysLTs: CysLT1, CysLT2, and GPR99^22^. However, BLT2 deficiency did not affect PLY-induced cysLTs production.

We next identified the cells responsible for PLY-dependent cysLTs production. LTC_4_ is produced from AA through 5-LO and LTC_4_ synthase, and is subsequently converted into LTD_4_ and then LTE_4_ extracellularly^23^. The most important and well-known source of LTC_4_ is mast cells; therefore, we treated bone marrow-derived mast cells (BMMCs) with PLY and measured the cysLTs concentrations in the culture supernatant. Extremely high amounts of LTC_4_ (~3 nmol/1 × 10^6^ BMMCs) were detected after PLY treatment (Fig. 4C), indicating that PLY stimulates both the production and release of cysLTs from mast cells, leading to vascular hyperpermeability and bronchoconstriction in the mouse lung. Previous reports demonstrate that BLT2 is expressed by mast cells^12^,^24^. To compare the functions of mast cells in BLT2-WT and BLT2-KO mouse lungs, we measured the expression of mRNA for mast cell markers, including *Hdc* (histidine decarboxylase), *Mcpt4* (chymase), *Mcpt6* (tryptase), *Cpa3* (carboxypeptidase), and *Kit* (stem cell factor receptor)^25^. None of these mast cell markers was affected by BLT2 deficiency (Fig. 4D), suggesting that mast cell maturation is intact in BLT2-KO mice. These data suggest that PLY stimulates the production of large amounts of cysLTs by mast cells in both BLT2-WT and BLT2-KO mice.

### 12-HHT/BLT2 signaling suppresses CysLT1 expression in vascular endothelial cells

**Figure 5:**
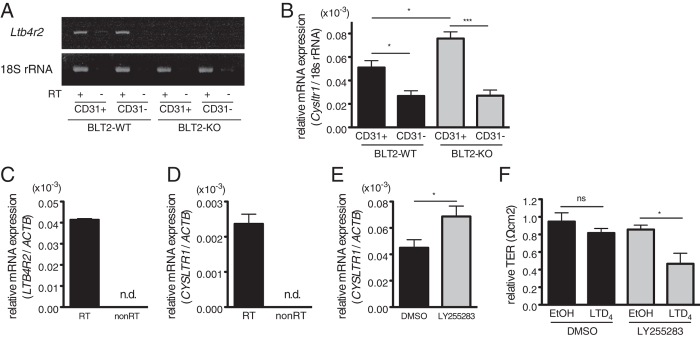
12-HHT/BLT2 signaling suppresses CysLT1 expression in vascular endothelial cells. (**A,B**) CD31-positive MLEC and CD31-negative cells were separated from mouse lung, and subjected to RT-PCR for BLT2 (**A**) and quantitative RT-PCR for CysLT1 (**B**) (n = 8–10). Data were analysed by one-way ANOVA, followed by Tukey’s *post-hoc* test: *p < 0.05; ***p < 0.001. (**C**,**D**) Quantitative RT-PCR for BLT2 (**C**) and CysLT1 (**D**) mRNA in HUVEC: n.d., not detected. (**E**) CysLT1 mRNA expression after 3 days of treatment with 1 μM LY255283 in HUVEC (n = 6). Data were analysed by Student’s *t* test: *p < 0.05. (**F**) HUVEC were pretreated with 1 μM LY255283 for 3 days. TER was measured after 5 min stimulation with vehicle (EtOH) or 1 μM LTD_4_ (n = 3). Data were analysed by one-way ANOVA, followed by Tukey’s *post-hoc* test: ns, not significant; *p < 0.05.

Because PLY-induced cysLTs production was comparable between BLT2-WT and BLT2-KO mice (Fig. 4A,B), we next examined the expression level of cysLTs receptor CysLT1. CD31-positive mouse lung endothelial cells (MLEC) were isolated and subjected to quantitative PCR. Expression of BLT2 mRNA was observed both in MLEC and CD31-negative cells from BLT2-WT mouse lung (Fig. 5A, Supplementary Fig. S1), consistent with the immunohistochemical analysis (Fig. 2). CysLT1 mRNA levels in MLEC were significantly higher in BLT2-KO than in BLT2-WT (Fig. 5B), though those in whole lung (data not shown) and in CD31-negative lung cells (Fig. 5B) from BLT2-WT and BLT2-KO mice were similar. Because measurement of Ca^2+^ mobilization or transepithelial electrical resistance (TER) with isolated primary MLEC was unsuccessful, we employed HUVEC, which endogenously express both BLT2 (Fig. 5C) and CysLT1 (Fig. 5D) for further analysis. The blockade of 12-HHT/BLT2 signaling by the BLT2 antagonist LY255283 induced CysLT1 mRNA expression (Fig. 5E) and decreased TER after LTD_4_ stimulation (Fig. 5F) in HUVEC. These data suggest that BLT2 deficiency enhances CysLT1 expression in vascular endothelial cells, resulting in the enhanced susceptibility to cysLTs produced by PLY administration in BLT2-KO mice.

### A CysLT1 antagonist improves PLY-induced ALI in BLT2 deficient mice

**Figure 6:**
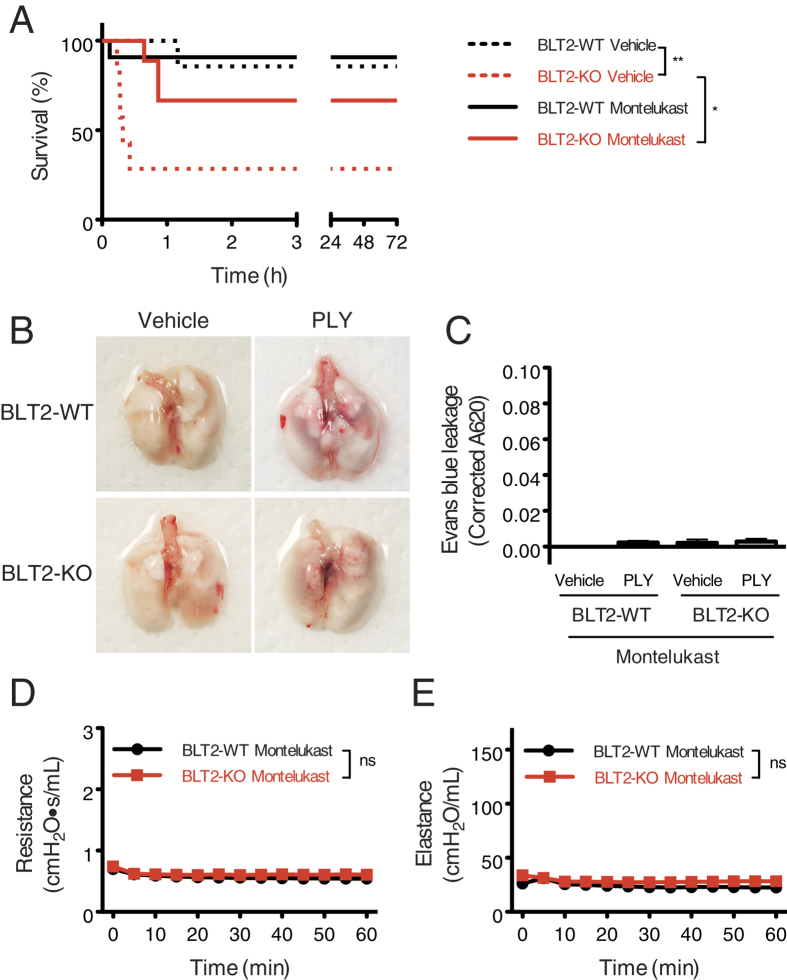
A CysLT1 antagonist ameliorates PLY-induced mortality, vascular permeability, and airway resistance in BLT2-deficient mice. (**A**) Montelukast (CysLT1 antagonist) or vehicle were administered by gavage at 24 h and 4 h prior to PLY treatment. Mice were then treated with PLY, and survival was monitored (n = 7–11). Data were analyzed using the log-rank test: *p = 0.032; **p = 0.0046. (**B**,**C**) Mice were treated with montelukast by gavage and injected with 0.5% Evans blue intravenously. The accumulation of Evans blue in tissues after administration of PLY was calculated as in Fig. 3 (n = 3 for vehicle; n = 5–7 for PLY treatment). (**D**,**E**) Montelukast-pretreated mice received PLY and were mechanically ventilated. The airway resistance (**D**) and the elastance (**E**) were measured every 5 min for 60 min (n = 6). Data were analyzed by two-way ANOVA.

CysLT1 is one of the receptors for cysLTs and is deeply involved in asthmatic responses such as bronchoconstriction and vascular leakage; thus, several CysLT1 antagonists are clinically used to treat bronchial asthma and allergic rhinitis^26^,^27^,^28^. To examine the association between cysLTs and PLY-dependent lethality, mice were treated with a CysLT1-selective antagonist, montelukast, by gavage at 24 and 4 h before intratracheal administration of PLY. The survival of montelukast-pretreated BLT2-KO mice was markedly higher than that of vehicle control mice (Fig. 6A). Of note, PLY-induced vascular leakage was completely abrogated in both BLT2-WT and BLT2-KO mice by pretreatment with montelukast (Fig. 6B,C). The PLY-dependent increases in airway resistance and elastance in BLT2-KO mice were also entirely ameliorated by pretreatment with montelukast (Fig. 6D,E). These results clearly indicate that CysLT1 is involved in PLY-induced ALI in BLT2-KO mice.

**Figure 7:**
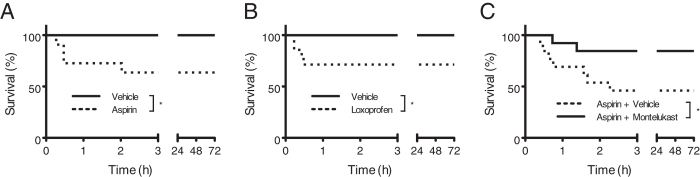
NSAIDs treatment aggravates mortality from PLY-induced ALI in WT mice. (**A**,**B**) BALB/c mice started to receive aspirin (**A**) 0.18 mg/mL) or loxoprofen (**B**) 30 μg/mL) in drinking water 2 days before intratracheal PLY treatment and monitored for survival until 72 h (n = 10–14). (**C**) BALB/c mice received aspirin in drinking water for 7 days and given montelukast by gavage twice prior to PLY treatment (n = 13). Kaplan-Meier curves are shown for each experimental condition. Data were analyzed using the log-rank test: *p < 0.05.

Finally we asked whether PLY-dependent mortality is affected by treatment of mice with NSAIDs which inhibit the production of the BLT2 ligand 12-HHT. As expected, pretreatment of WT mice with aspirin and loxoprofen increased mortality by PLY administration (Fig. 7A,B, respectively). Moreover, montelukast treatment also improved the survival ratio of aspirin-pretreated mice after PLY administration (Fig. 7C). These results emphasize that 12-HHT/BLT2 signaling plays important role in protecting against PLY-related ALI, possibly by decreasing CysLT1 expression.

## Discussion

In this study, we explored the effects of BLT2-deficiency on the acute lung injury using BLT2-KO mice. BLT2 was originally cloned as a highly homologous receptor to BLT1, the receptor for a potent chemoattractant LTB_4_^16^, with 45% on amino acid level, and its gene locus is overlapped on the promoter region of the BLT1 gene^11^. As extremely high amount of LTB_4_ is required to activate BLT2, we explored the endogenous ligand and identified the 12-HHT as a high-affinity endogenous ligand for BLT2, which had long been assumed as a functionless by-product of thromboxane A_2_ synthesis^12^. 12-HHT is massively produced *via* thromboxane A_2_ synthase (TxA_2_S) in association with blood coagulation, but is also constantly produced in a TxA_2_S-independent fashion under the presence of COXs^21^. Previously we reported that 12-HHT/BLT2 axis plays biological roles in maintaining intestinal barrier function^14^ and in accelerating skin wound healing^13^, and that BLT2 enhances the expression of tight junctional proteins^15^. Based on these evidences, we investigated whether BLT2 plays biophylactic ability in the lung, which is also exposed to outer environment, using a pneumococcal toxin PLY.

Of particular interest is that, although BLT2 deficiency caused PLY-dependent sudden death, WT mice rarely died; this suggests that inhibiting BLT2 signaling critically exacerbates PLY-induced ALI. Although there is no report on BLT2 deficiency in human, several reports suggest the relationship between pneumococcal infection and NSAIDs in human. Recent reports show that NSAIDs given during the early stages of lower respiratory tract infections may aggravate serious pneumococcal pneumonia in human patients^29^,^30^. In addition, a murine model revealed the detrimental role of aspirin in experimental pneumococcal infection^31^. Because the BLT2 ligand 12-HHT is produced from AA *via* COXs, its production is completely inhibited by NSAIDs^13^,^21^. Consistent with these previous reports, pretreatment with aspirin or loxoprofen certainly aggravated the survival ratio of PLY-treated mice. These data explain the possible molecular mechanisms underlying the finding that patients taking NSAIDs exhibit more severe symptoms of pneumococcal pneumonia^29^,^30^. Furthermore, clinicians occasionally encounter ALI flare-ups during a course of antibiotics, possibly due to the release of large amounts of PLY from dead bacteria. Here, we also show that the CysLT1 antagonist blocks PLY-dependent ALI both in BLT2-KO mice and in NSAIDs-pretreated mice, suggesting that the CysLT1 antagonist is a candidate drug for treating severe ALI in patients with pneumococcal pneumonia.

Although the comparable levels of PLY-dependent cysLTs production were observed in BLT2-WT and BLT2-KO mice, the expression of CysLT1 mRNA in MLEC was enhanced in BLT2-KO mice. Consistently, pharmacological inhibition of BLT2 signaling by LY255283 increased the mRNA expression of CysLT1 and subsequently enhanced the LTD_4_-dependent barrier dysfunction in HUVEC. Recent reports show that CysLT1 signaling is negatively regulated by several GPCRs, including CysLT2 and GPR17^32^,^33^,^34^. These receptors interact with CysLT1, thereby inhibiting LTD_4_-dependent signaling. Our findings suggested that BLT2 negatively regulates CysLT1 function in lung, by suppressing CysLT1 expression; hence, BLT2 deficiency augments cysLTs-CysLT1-dependent bronchoconstriction and vascular leakage, which in turn results in acute respiratory failure and sudden death in BLT2-KO mice after PLY administration.

Although the CysLT1 antagonist completely inhibited PLY-dependent vascular leakage and bronchoconstriction, it did not completely protect all BLT2-KO mice from death, indicating that alternative mechanisms of PLY-induced lethality may be in play. One possibility is that epithelial fragility may occur in BLT2-KO mouse lung; indeed, a recent report showed that BLT2 augments tight junctions^15^. BLT2 expressed in AT II cells played no role in surfactant production; however, the role of BLT2 in other AT II cell functions (e.g., as progenitors of AT I cells) is unknown. Another possibility would be the involvement of other lipid mediators. Recently, a growing number of reports describe a relationship between specialized pro-resolving lipid mediators (SPMs) and acute lung inflammation^35^. Although we failed to detect several SPMs (including lipoxin A_4_, protectin D1, and resolvins) in BAL fluid in the presence/absence of PLY (data not shown), other SPMs such as maresin 1^36^ might be involved in lethal ALI.

Collectively, the data reported herein reveal a novel role for BLT2 in protecting lung tissues in addition to its previously reported role in the intestine and skin. Thus, BLT2 plays a crucial role in tissues exposed to the external environment, raising the possibility that a CysLT1 antagonist may protect patients with pneumococcal pneumonia from ALI.

## Methods

### Mice

BLT2-KO (*Ltb4r2*^−/−^) and BLT1-KO (*Ltb4r1*^−/−^) mice were generated as described previously^14^,^37^ and backcrossed with BALB/c or C57BL/6 mice over 12 generations. The mice used for the experiments were 10–14 weeks old. All animals were bred in specific pathogen-free conditions.

### Ethics statement

All animal experiments were approved by the Ethical Committee for Animal Experiments in Kyushu University and Juntendo University. All the methods in this manuscript were carried out in accordance with the approved guidelines and regulations.

### Purification of recombinant PLY

Recombinant PLY was expressed in *E. coli* transformed with plasmid DNA encoding 6 × His-tagged PLY and purified as described previously^38^. Briefly, the PLY plasmid was introduced into *E. coli* strain M15[pREP4] (QIAGEN), and the bacteria were cultured at 30 °C for 18 h. IPTG was then added to a final concentration of 0.1 mM to induce PLY expression. After 5 h of induction, cells were collected and lysed with X Tractor buffer® (Clontech) containing 2 unit/mL DNase I (Roche) and a protease inhibitor cocktail (nacalai). The lysate was centrifuged at 10,000 × g for 20 min, and the supernatant was collected and applied to TALON® metal affinity resin (Clontech). Recombinant PLY was eluted with elution buffer (50 mM Tris-HCl, pH 7.4, 150 mM NaCl, and 200 mM Imidazole) and then dialyzed against saline.

### Murine model of ALI

Mice were anesthetized by intraperitoneal injection of ketamine (Daiichi-Sankyo; 100 mg/kg) and xylazine (SIGMA; 10 mg/kg), and oro-tracheally intubated with 20-gauge intravenous catheters (TERUMO). PLY (50 ng) or an equivalent volume of saline (50 μL; as vehicle) were injected through the catheter and allowed to absorb into the alveoli by spontaneous respiration. Mice were then monitored for 72 h to evaluate survival. For the NSAIDs-pretreatment experiments, mice were started to receive aspirin (SIGMA; 0.18 mg/mL in drinking water) or loxoprofen (Tokyo Chemical Industry; 30 μg/mL in drinking water) 2 days or 7 days before PLY injection as reported^13^,^39^. For experiments with the CysLT1 antagonist, mice were pretreated with montelukast (CAYMAN) as previously reported, with a minor modification^40^. In short, montelukast (5 mg/kg) was suspended in 1% methylcellulose and administered by gavage twice, at 1 d and 4 h prior to PLY treatment.

### Analysis of BAL fluid

BAL was performed 10 min after PLY administration. One milliliter of PBS containing 2 mM EDTA was infused into the lungs through the intubated catheter, and 700 μL of the solution was collected as BAL fluid. The total protein concentration, albumin concentration, and LDH activity in the BAL fluid were measured using Protein Assay Bicinchoninate kit (nacalai), Mouse Albumin ELISA Quantitation Set (Bethyl Laboratories), and LDH Cytotoxicity Detection Kit (Takara), respectively, according to the manufacturers’ protocols. The concentrations of eicosanoids were measured by LC-MS/MS as described previously^21^. The PC concentration was measured in an enzyme-based fluorescent assay as previously reported^18^.

### Vascular permeability assay

The permeability of the lung vasculature was measured according to the accumulation of intravenously injected Evans blue in the tissues as described previously^41^,^42^, with a minor modification. Briefly, mice were intravenously injected with 100 μL of 0.5% Evans blue (nacalai) at 30 min prior to PLY administration. The lungs were then perfused with saline and harvested 10 min after PLY treatment, followed by snap freezing in liquid nitrogen. Lungs were then homogenized in 2 mL of PBS. Evans blue was extracted from the lung homogenate by adding twice the volume of formamide and incubating at 60 °C for 18 h. Samples were centrifuged at 12,000 × g for 30 min. The concentration of Evans blue in the supernatants was then measured at the absorbance of 620 and 740 nm using a dual wavelength spectrophotometric method. The concentration (as represented by the corrected A620 value that omits contaminating heme pigments) was calculated using the following formula: Corrected A620 = A620 − (1.426 × A740 + 0.03).

### Immunohistochemistry of mouse lung

Mouse lungs were perfused with 10 mL of saline (through the right ventricle), harvested, and fixed overnight with 4% paraformaldehyde. Fixed samples were embedded in Tissue-Tek® O.C.T. compound (SAKURA Finetek) and serially sectioned (3 μm thickness). The tissues were incubated in 0.3% H_2_O_2_/methanol for 30 min to block endogenous peroxidase activity. The sections used for CD31 staining were heat-treated in 0.01 M citrate buffer (pH 6.0) for antigen retrieval before peroxidase blocking. Tissues were blocked with 5% goat serum and incubated with appropriate primary antibodies overnight at 4 °C, followed by secondary antibodies for 60 min at room temperature. The following primary antibodies were used: rabbit anti-mouse BLT2 (5 μg/mL; generated in our laboratory by immunizing a rabbit with a BLT2 C-terminal peptide); rabbit anti-proSP-C (1:1000; Millipore); hamster anti-T1α (1:2000; BioLegend); rabbit anti-CD31 (1:50; abcam), rabbit IgG (5 μg/mL; DAKO); and hamster IgG (1:2000; BioLegend). The secondary antibodies were biotinylated anti-rabbit goat IgG (1:300; DAKO) and biotinylated anti-hamster goat IgG (1:200; Vector Laboratories). Tissues were labeled with horseradish peroxidase-conjugated streptavidin (DAKO), and the signals were detected by incubating with diaminobenzidine. Images were captured under a microscope (Keyence BZ9000).

### Quantitative RT-PCR

Mouse AT II cells were isolated as previously reported^43^. In brief, lungs were perfused with saline, harvested, and digested with Dispase II (Roche). Cells were separated from the bronchi, minced in DMEM (Wako) containing 25 mM HEPES and 0.1 mg/mL DNase I (Roche), serially passed through 100 (BD Falcon), 40 (BD Falcon), and 20 (PARTEC) μm cell strainers, and subjected to magnetic cell sorting (MACS; Miltenyi Biotec). CD16/32- and CD45-positive cells were removed by negative selection. MLECs were isolated according to previous report with minor modifications^44^. Briefly, lungs were minced and digested with Dispase II (Roche) and Collagenase D (Roche), separated to single cell suspension by passing through 70 μm cell strainer (BD Falcon), and subjected to MACS. CD31-positive cells were collected as MLEC. Anti-CD16/32, anti-CD45, anti-CD31 antibodies were purchased from BD pharmingen. Total RNA was isolated from AT II cells, MLEC, HUVEC or mouse lungs using TRIzol® Reagent (Life Technologies) and reverse transcribed with SuperScript III First-Strand Synthesis System for RT-PCR (Invitrogen), according to the manufacturers’ protocol. The target genes were detected by real-time PCR in a LightCycler (Roche). The following primers were used: *Sftpa* forward, 5′-TTCCAGGGTTTCCAGCTTACCT-3′ and reverse, 5′-AGTTGACTGACTGCCCATTGGT-3′; *Sftpb* forward, 5′-TGGAACACCAGTGAACAGGCTA-3′ and reverse, 5′-GCATGTGCTGTTCCACAAACTG-3′; *Sftpc* forward, 5′-TGATGGAGAGTCCACCGGATTA-3′ and reverse, 5′-CCTACAATCACCACGACAACGA-3′; 18S rRNA forward, 5′-CGGCTACCACATCCAAGGAA-3′ and reverse, 5′-GCTGGAATTACCGCGGCT-3′; *Cysltr1* forward, 5′-CCTCTCCGTGTGGTCTATTATGT-3′, and reverse, 5′-ACCGGAAAAAGCTCATGGCT-3′; *CYSLTR1* forward, 5′-TGCAGAAGTCCGTGGTCATA-3′, and reverse, 5′-GGAGAGGGTCAAAGCAACAA-3′; *ACTB* forward, 5′-TGGCACCCAGCACAATGAA-3′, and reverse, 5′-CTAAGTCATAGTCCGCCTAGAAGCA-3′. Primers specific for *Ltb4r2*, *LTB4R2*, *Hdc*, *Mcpt4*, *Mcpt6*, *Cpa3*, *Kit*, and *Gapdh* were described previously^13^,^25^.

### H&E staining

BLT2-KO mouse lungs were harvested immediately after death. WT mice were sacrificed at the time of BLT2-KO mouse death caused by PLY. Lungs were collected, fixed in 10% formalin, paraffin-embedded, sectioned, and stained with H&E.

### Mast cell stimulation

BMMCs were prepared in IL-3-containing medium, as described previously^25^. Briefly, 1 × 10^6^ cells were suspended in 200 μL of HBSS containing 0.1% bovine serum albumin, and then stimulated with PLY (50 ng/mL) for 5 min. The eicosanoid content in the supernatant was then measured by LC-MS/MS.

### Pulmonary function measurement

Mice were anesthetized with ketamine and xylazine and then intubated with metal 18-gauge catheters *via* a tracheotomy. PLY was administered through the catheter and allowed to absorb into the alveoli by spontaneous respiration. After administration of PLY, anesthetic agents were added to stop spontaneous breathing. Mice were then immediately connected to the flexiVent™ (SCIREQ), followed by measurement of dynamic resistance and elastance every 5 min for 60 min.

### TER measurement

HUVECs were purchased from Lonza and cultured in EGM™ BulletKit™ Medium (Lonza). TER was measured as previously reported^15^,^45^. Briefly, cells were seeded at 1 × 10^5^ cells/well on fibronectin (25 μg/mL)-coated membrane inserts (Millicell® Cell Culture Inserts; Millipore) in culture medium, supplemented with 1 μM LY255283 or 0.01% DMSO as vehicle control, and cultured for 3 days to achieve confluency. TER was measured before and 5 min after 1 μM LTD_4_ stimulation, using Millicell-ERS-2 volt-ohmmeter (Millipore). The value was calculated with the following formula: TER (Ωcm^2^) = (R sample − R blank) × effective membrane area, and standardized by the basal TER value.

### Statistics

Data are expressed as the mean ± SEM. Survival data were analyzed using the log-rank test. Two data sets were compared using a two-tailed Student’s *t* test. ANOVA was used for multiple comparisons. *P* values < 0.05 were considered statistically significant. All data were calculated using Prism version 5.0 (GraphPad Software).

## Additional Information

**How to cite this article**: Shigematsu, M. *et al.* Leukotriene B_4_ receptor type 2 protects against pneumolysin-dependent acute lung injury. *Sci. Rep.* **6**, 34560; doi: 10.1038/srep34560 (2016).

## Supplementary Material

Supplementary Information

## Figures and Tables

**Table 1:** Eicosanoid contents of BAL fluid and lung tissues.

Upper: in BAL fluid (pM)	BLT2^+/+^		BLT2^−/−^	
Lower: in lung (pmol/mg)	Vehicle	PLY	Vehicle	PLY
LTB_4_	1.30 ± 0.23	248.53 ± 15.07	1.24 ± 0.24	175.82 ± 8.22
	0.000 ± 0.00	0.003 ± 0.00	0.000 ± 0.00	0.003 ± 0.00
LTC_4_	20.08 ± 2.92	4212.13 ± 268.37	129.40 ± 11.70	3039.29 ± 122.15
	0.000 ± 0.00	0.097 ± 0.01	0.000 ± 0.00	0.111 ± 0.01
LTD_4_	13.07 ± 2.72	3893.46 ± 190.74	19.55 ± 4.10	3337.15 ± 172.69
	0.000 ± 0.00	0.007 ± 0.00	0.000 ± 0.00	0.008 ± 0.00
LTE_4_	11.97 ± 1.90	3151.79 ± 221.73	59.68 ± 15.44	2713.18 ± 216.99
	0.000 ± 0.00	0.008 ± 0.00	0.000 ± 0.00	0.006 ± 0.00
PGE_2_	88.81 ± 5.24	100.71 ± 4.74	82.83 ± 5.54	109.89 ± 5.57
	0.018 ± 0.00	0.014 ± 0.00	0.010 ± 0.00	0.010 ± 0.00
PGD_2_	24.10 ± 1.40	27.16 ± 0.96	15.09 ± 0.81	31.88 ± 1.71
	0.004 ± 0.00	0.005 ± 0.00	0.003 ± 0.00	0.004 ± 0.00
PGF_2_α	26.67 ± 2.88	43.71 ± 2.38	15.18 ± 0.88	50.70 ± 3.61
	0.003 ± 0.00	0.003 ± 0.00	0.002 ± 0.00	0.003 ± 0.00
12-HHT	10.41 ± 1.51	8.24 ± 0.62	11.89 ± 1.14	22.46 ± 2.50
	0.018 ± 0.00	0.026 ± 0.00	0.014 ± 0.00	0.015 ± 0.00
12-HETE	268.55 ± 43.07	687.77 ± 32.73	163.22 ± 15.66	445.54 ± 17.67
	0.061 ± 0.01	0.039 ± 0.00	0.019 ± 0.00	0.050 ± 0.01
15-HETE	31.37 ± 5.48	25.48 ± 1.65	26.41 ± 3.34	20.38 ± 2.29
	0.010 ± 0.00	0.006 ± 0.00	0.001 ± 0.00	0.004 ± 0.00

BAL fluid and lung tissues were collected 10 min after PLY administration. The eicosanoid content was measured by LC-MS/MS (n = 4–11). Upper rows, concentration in BAL fluid (pM); lower rows, concentration in lung tissue (pmol/mg tissue).
