# Supplementary material for: Leukotriene B4 receptor type 2 protects against pneumolysin-dependent acute lung injury
Source: Sci Rep. 2016 Oct 5;6:34560. doi: 10.1038/srep34560 (PMC5050523; doi:10.1038/srep34560)

Supplementary information

Leukotriene B_4 receptor type 2 protects against pneumolysin-dependent acute lung injury

Misako Shigematsu[1,2], Tomoaki Koga[1], Ayako Ishimori[3], Kazuko Saeki[1], Yumiko Ishii[4], Yoshitaka Taketomi[5], Mai Ohba[1], Airi Jo-Watanabe[1], Toshiaki Okuno[1], Norihiro Harada[3], Takeshi Harayama[6,7], Hideo Shindou[6], Jian-Dong Li[8], Makoto Murakami[5], Sumio Hoka[2], Takehiko Yokomizo[1*]

[1]Department of Biochemistry, Juntendo University School of Medicine, Tokyo, Japan.

[2]Department of Anesthesiology and Critical Care Medicine, Graduate School of Medical Sciences, Kyushu University, Fukuoka, Japan.

[3]Department of Respiratory Medicine, Juntendo University School of Medicine, Tokyo, Japan.

[4]Research Institute for Diseases of the Chest, Graduate School of Medical Sciences, Kyushu University, Fukuoka, Japan.

[5]Lipid Metabolism Project, Tokyo Metropolitan Institute of Medical Science, Tokyo, Japan.

[6]Lipid Signaling Project, National Center for Global Health and Medicine, Tokyo, Japan.

[7]Department of Biochemistry, University of Geneva, Geneva, Switzerland.

[8]Center for Inflammation, Immunity and Infection, Institute for Biomedical Sciences, Georgia State University, Atlanta, GA.

*Correspondence should be addressed to T.Y. (yokomizo-tky@umin.ac.jp).

Figure 5A *Ltb4r2*

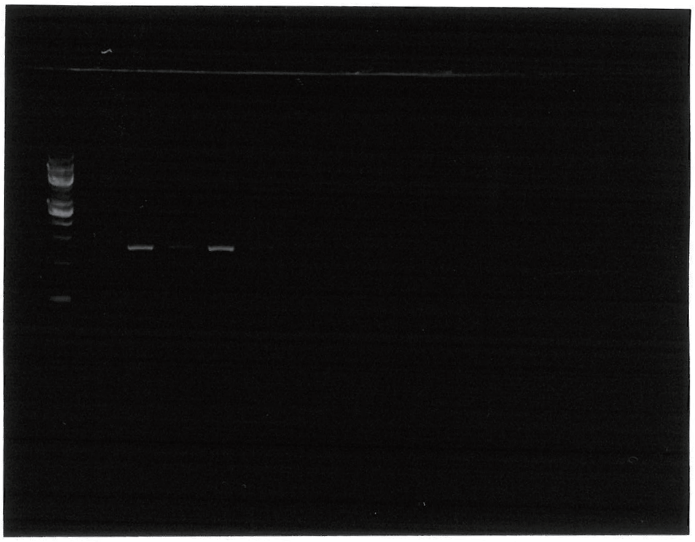

Figure 5A 18S rRNA

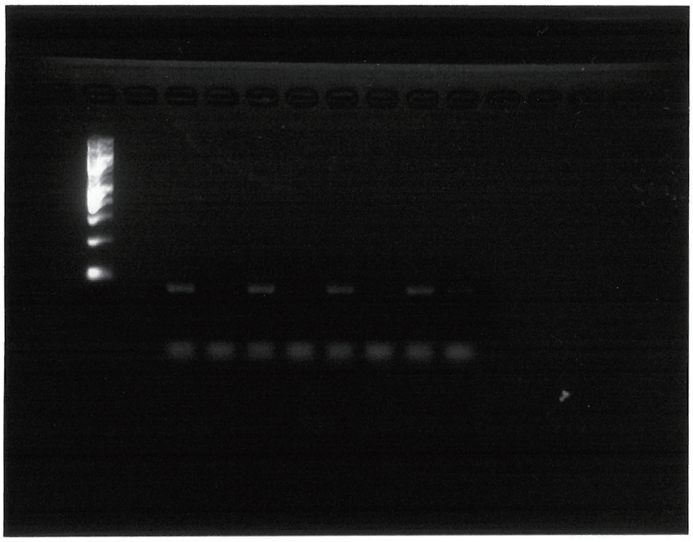

Supplement: Supplementary Information [file srep34560-s1.pdf]
